# Solution growth and thermal treatment of crystals lead to two new forms of 2-((2,6-dimethylphenyl)amino)benzoic acid†

**DOI:** 10.1039/c7ra13353g

**Published:** 2018-04-24

**Authors:** Rong Hu, Yunping Zhoujin, Meng Liu, Mingtao Zhang, Sean Parkin, Panpan Zhou, Jianzhi Wang, Faquan Yu, Sihui Long

**Affiliations:** Key Laboratory for Green Chemical Process of Ministry of Education, School of Chemical Engineering and Pharmacy, Wuhan Institute of Technology 693 Xiongchu Road Wuhan Hubei 430073 China fyuwucn@gmail.com sihuilong@wit.edu.cn longsihui@yahoo.com +86 02787194980 +86 15549487318; Computational Center for Molecular Science, College of Chemistry, Nankai University Tianjin China; Department of Chemistry, Lanzhou University Lanzhou Gansu China; Department of Chemistry, University of Kentucky Lexington Kentucky USA

## Abstract

We report the discovery of two new forms (II and III) of a potential non-steroidal anti-inflammatory and thyroid drug, 2-((2,6-dimethylphenyl)amino)benzoic acid (HDMPA) through solution growth and thermal treatment of crystals. Form II has been discovered through crystal growth in a variety of solvents, and characterized by single-crystal X-ray diffraction, powder X-ray diffraction (PXRD), FT-IR, and Raman spectroscopy. Form II converts into form III upon thermal treatment, as indicated by the phase behavior study of form II with differential scanning calorimetry (DSC). Form III has been characterized by IR, Raman and PXRD. Conformational flexibility of the molecule seems to lead to the polymorphism of the system. A conformational scan shows the conformational minima correspond to the conformers in the polymorphs. Lattice energy calculations show energies of −48.14 and −50.31 kcal mol^−1^ for forms I and II, providing information on the relative stability for each form. Hirshfeld analysis revealed that intermolecular interactions such as C⋯C, H⋯H, C⋯H, and H⋯O contribute to the stability of the crystal forms.

## Abstract 2

Two new forms of a potential non-steroidal anti-inflammatory and thyroid drug, 2-((2,6-dimethylphenyl)amino)benzoic acid were discovered and investigated experimentally and theoretically.

## Graphical abstract

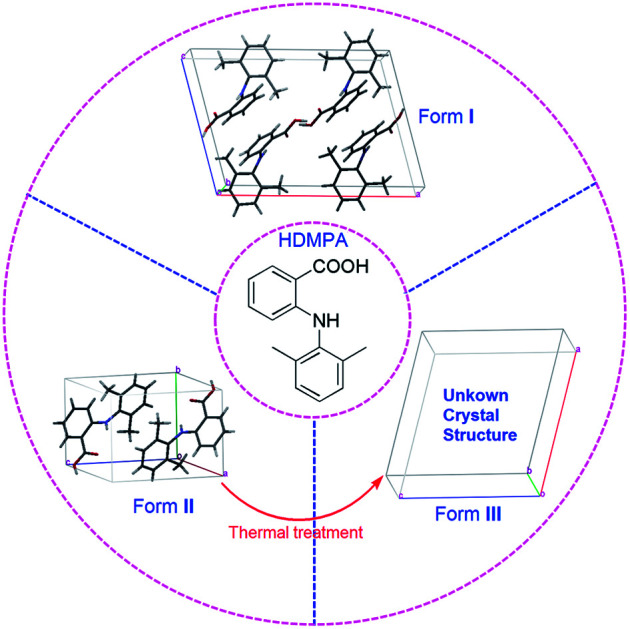

## 1. Introduction

*N*-Anthranilic acids, the nitrogen isosteres of salicylic acid, constitute an important class of non-steroidal anti-inflammatory drugs (NSAIDs).^1^ Classic anthranilic acid NSAIDs include mefenamic acid, meclofenamic acid, chlofenamic acid, and other structurally related compounds.^2^ Their medical application is not limited to NSAIDs, and they can also be used as analgesics and antirheumatics.^1^ Recently, this class of compounds have been investigated as therapeutics for neurodegenerative and amyloid diseases as well as cancer, and some have shown great promise.^3–5^ At the same time, these compounds can be used as ligands in inorganic complexes in the search for anticancer drugs.^6^ In addition, fenamic acids are also synthetic precursors to acridones and acridines, which present bioactive properties such as anti HIV, anticancer, antibacterial, antifungal, and antimalarial activities.^7^

Likely due to their intrinsic conformational flexibility (as diarylamines), *N*-anthranilic acids tend to be polymorphic, *i.e.*, existing in more than one crystal form. Several important *N*-anthranilic acid NSAIDs have multiple crystal forms. For example: two, five, nine, two, and four forms have been found for mefenamic acid,^8^ tolfenamic acid,^9^ flufenamic acid,^10^ niflumic acid,^11^ and clonixin,^12^ respectively. The existence of nine forms of flufenamic acid is reported to be the current world record for most forms having been structurally characterized by single-crystal X-ray diffraction. Recently, clonixin was found to form a solvate with *N*,*N*-dimethylformamide.^13^ Polymorphism is important both theoretically (CSP)^14,15^ and practically.^16–19^ Polymorphism is of particular significance in pharmaceuticals because different forms of the same API (Active Pharmaceutical Ingredient) may have different kinetic, thermodynamic, surface, mechanical, and packaging properties, which can affect clinical formulation and eventual bioavailability.^20,21^

HDMPA is an *N*-anthranilic acid similar to other NSAIDs with potent anti-inflammatory properties.^22^ In addition, it has ramifications in the treatment of disorders caused by thyroid hormone excess by inhibiting triiodothyronine uptake by the hepatocytes.^23^

In 2004, in an attempt to synthesize phenylorganotin complexes as anti-tuberculosis drugs, Dokorou *et al.* discovered the first crystal structure of HDMPA (designated form I). The asymmetric unit of form I consists of two crystallographically independent molecules (*Z*′ = 2) and the two molecules are distinct in geometry.^24^

Our lab has been interested in the polymorphism of diarylamines for the past decade. We have systematically investigated a series of compounds using a combination of both experimental and theoretical approaches.^25–29^ Recently we also studied the polymorphism of an important *N*-anthranilic acid, 4-chloro-phenylanthranilic acid and highlighted the role played by both the conformational flexibility and sp^2^ C–H⋯Cl hydrogen bond.^30^

In this study, we report the discovery and characterization of a 2nd form of HDMPA, as well as the structural comparison of forms I and II, and the generation and characterization of a third form (III) of the compound, and the corresponding theoretical investigation to shed light on the polymorphism of this system.

## 2. Experimental section

### 2.1. General

All solvents and reagents were purchased from commercial sources and used as received: 2-chlorobenzoic acid was from J&K Chemical (Beijing, China), 2,6-dimethylaniline was from Energy Chemical (Shanghai, China), and Cu_2_O was from Aladdin (Shanghai, China); Cu, K_2_CO_3_, 2-ethoxyethanol, and the solvents used for crystal growth were from Sinopharm Chemical Reagent Co., Ltd (Shanghai, China). The IR and Raman spectra were recorded using an FT-IR Perkin-Elmer LX10-8873 and a Thermo Electron DXR Laser Confocal Microscopy Raman Spectrometer, respectively. NMR spectra were obtained on a Varian INOVA spectrometer at an observation frequency of 400 MHz. Thermal analyses were performed on an SII DSC 6220 (SEIKO, Japan) apparatus, using a heating rate of 10 °C min^−1^.

### 2.2. Synthesis and characterization

#### 2.2.1 Synthesis of 2-((2,6-dimethylphenyl)amino)benzoic acid^31^

**Scheme 1:**
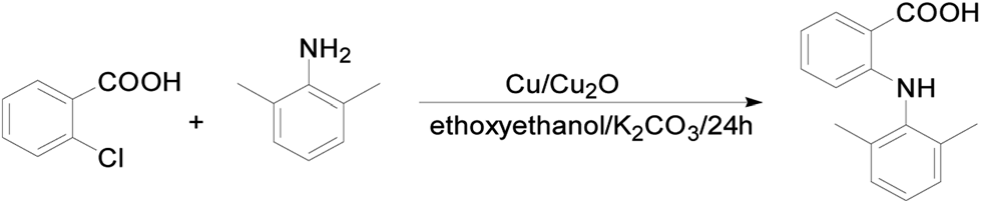
Synthesis of HDMPA.

2-Chlorobenzoic acid (1.38 g, 8.8 mmol), 2,6-dimethyl-phenylamine (1.13 g, 9.3 mmol), Cu (50.0 mg, 0.8 mmol), Cu_2_O (57.2 mg, 0.4 mmol) and K_2_CO_3_ (1.25 g, 8.8 mmol) were added to a round-bottom flask, followed by addition of 2-ethoxyethanol (3 mL) under nitrogen protection. The resulting mixture was refluxed at 130 °C for 24 h. The hot reaction mixture was poured into 30 mL of room temperature water. The insoluble components were removed by filtration. The crude product was obtained by precipitation upon acidification of the filtrate with dilute HCl. The product was purified on a silica-gel column using petroleum ether/ethyl acetate (v/v 100 : 1, *R*_f_ = 0.50) as eluent. The product was obtained as colorless solid (230 mg, yield%: 11) (Scheme 1).

^1^H-NMR (400 MHz, CDCl_3_): *δ* ppm 8.90 (s, 1H), 8.05 (d, 1H), 7.26 (t, 1H), 7.16 (s, 3H), 6.67 (t, 1H), 6.29 (d, 1H), 2.23 (s, 6H); ^13^C-NMR (100 MHz, CDCl_3_): *δ* ppm 174.2, 150.7, 136.9, 135.6, 132.5, 129.1, 128.0, 127.3, 128.3, 115.8, 112.7, 108.7, 18.2; IR (KBr, cm^−1^) 3346 (s), 3018–2539 (m), 1651 (s), 1578 (s), 1498(s), 1417 (s), 1250 (s), 1170 (s), 923 (m), 785 (s), 741 (s); MS (ESI): 242.1 (M + 1); mp: 208 °C.

### 2.3. Crystal growth

Slow evaporation was applied for polymorph screening of HDMPA.^32^ The experiment is as follows: pure HDMPA was dissolved in eighteen different solvents (the compound is insoluble in water), forming saturated solutions at ambient temperature (∼22 °C) (Table 1). The solutions were set to evaporate in a vibration-free environment until single crystals appeared or no solvent remained. For example, 50 mg of HDMPA was suspended in 5 mL HPLC grade acetonitrile. The mixture was agitated overnight and the remaining solid was pipette filtered. A vial containing the saturated solution was covered with perforated parafilm. Slow evaporation led to single crystals in about a week. Quench cooling was used to obtain form I, as reported in the literature. For quench cooling, a supersaturated solution of HDMPA in a mixture of THF and acetone (1 : 1 ratio) at 50 °C was rapidly cooled in a freezer of −20 °C. All crystallization experiments were conducted in an unmodified atmosphere. Each experiment was repeated multiple times. All experiments led to crystal form II. The crystals were identified by single-crystal X-ray diffraction when high-quality single crystals were available, and by powder X-ray diffraction for tiny crystals.

**Table 1:** Crystal growth of HDMPA in different solvents

Solvents	Method	Form
Acetone	Slow evaporation	II
Chloroform	Slow evaporation	II
Ethyl acetate	Slow evaporation	II
Methanol	Slow evaporation	II
Dichloromethane	Slow evaporation	II
Ethanol	Slow evaporation	II
Hexane	Slow evaporation	II
Pet ether	Slow evaporation	II
Acetonitrile	Slow evaporation	II
Ether	Slow evaporation	II
iso-Propanol	Slow evaporation	II
Dimethyl sulfoxide	Slow evaporation	II
Tetrahydrofuran	Slow evaporation	II
Dimethylformamide	Slow evaporation	II
Toluene	Slow evaporation	II
Benzene	Slow evaporation	II
Acetic acid	Slow evaporation	II
Tetrahydrofuran & acetone (1 : 1)	Slow evaporation	II
Tetrahydrofuran & acetone (various ratios)	Quench cooling	II

### 2.4. Crystal structure determination

The crystal structures of form II HDMPA were determined by single-crystal X-ray diffraction at both 90 K (low temperature, LT) and 296 K (room temperature, RT).

Data collection was carried out at 90 K on a Nonius kappaCCD diffractometer with MoKα radiation (*λ* = 0.71073 Å),^33^ and at 296 K on a Bruker SMART APEX II diffractometer.

Cell refinement and data reduction were done using SCALEPACK and DENZO-SMN for the low temperature structure,^34^ and SADABS and Bruker SMART for the room temperature structure. Structure solution and refinement were carried out using the SHELXS and SHELXL2016 programs, respectively.^35,36^

Powder X-ray diffraction (PXRD) data for each sample were collected on a Rigaku X-ray diffractometer with CuKα radiation (40 kV, 40 mA, *λ* = 1.5406 Å) between 5.0–50.0° (2*θ*) at ambient temperatures. The finely ground sample was placed on a quartz plate in an aluminum holder.

### 2.5. Thermal analyses

Phase behavior for the solid forms were studied by differential scanning calorimetry (DSC), *vide supra*.

### 2.6. Spectroscopic studies

For IR spectrum recording, samples were dispersed in KBr pellets. For Raman measurement, samples were compressed in a gold-coated sample holder.

### 2.7. Computational details

#### 2.7.1 Conformation search

**Fig. 1:**
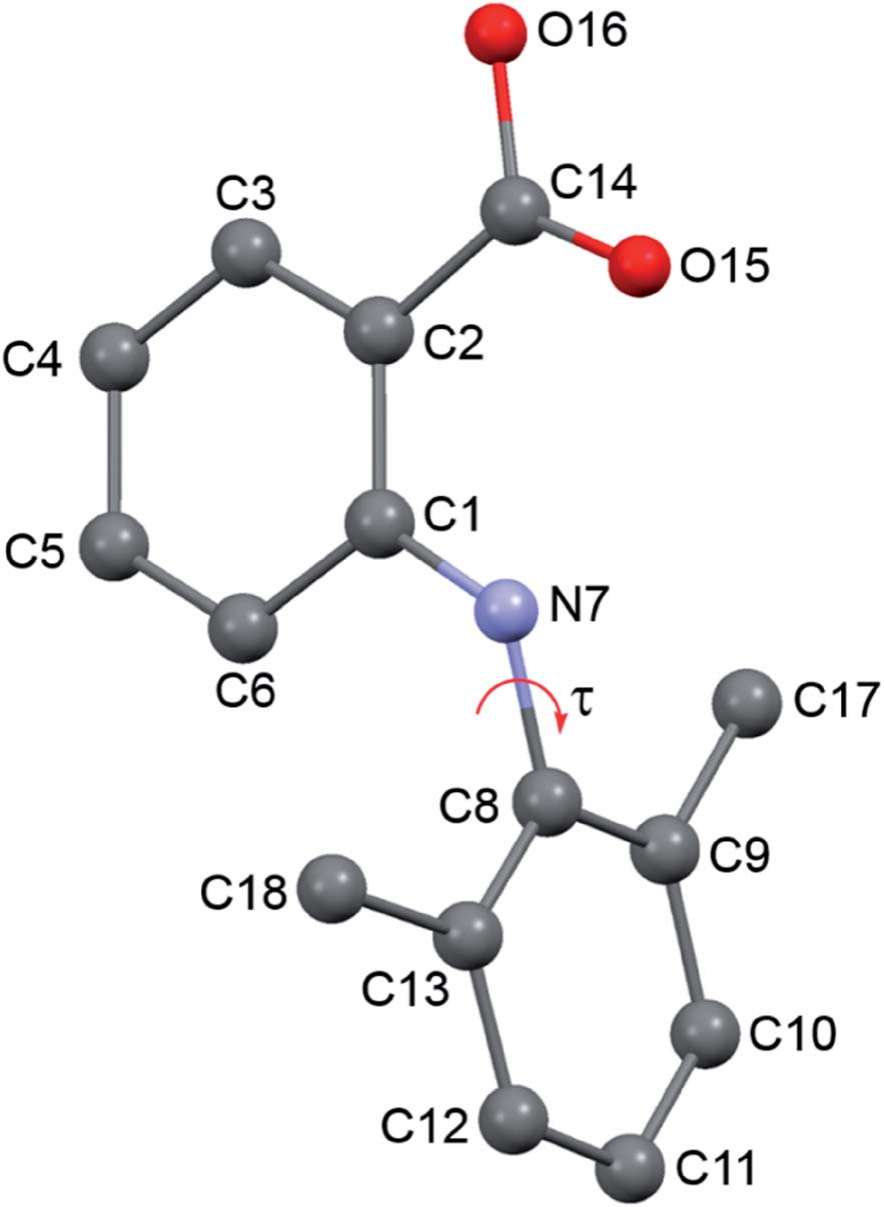
Labeling of HDMPA.

Based on the structure, bonds C2–C14, C1–N7 and N7–C8 (Fig. 1) are potential causes of the conformational flexibility. Nevertheless, we only evaluated the energy of different conformations of a single HDMPA molecule defined by the torsion angle C1–N7–C8–C9 (*τ*), with Gaussian 09 (Gaussian, Inc., Wallingford, CT), because C1–N7 has a bond length of ∼1.38 Å, indicating a double bond (which precludes rotation), and the carboxylic acid group in all conformers is coplanar with the aromatic ring in spite of the single-bond character for C2–C14 bond (C–C = ∼1.47 Å) (Table 2). The molecule was optimized from various initial structures in order to identify the most stable conformation, which was then used for scanning each torsion angle with all bond lengths, bond angles, and other torsion angles fixed. The B3LYP/6-311+G(d, p) level of theory was applied for the structural optimization and conformational search.

**Table 2:** Bond length of C1–N7 and N7–C8 in the conformers of HDMPA

Conformer	Bond length (Å)	
	C1–N7	N7–C8
I-A	1.380	1.432
I-B	1.363	1.465
II (RT)	1.375	1.441
II (LT)	1.378	1.441

#### 2.7.2 Lattice energy

To calculate the lattice energy for each HDMPA form, we first optimized the experimental crystal structures with the lattice parameters fixed. The optimization was conducted by DFT using the PW1PW functional with 6-21g** basis sets by Crystal14.^37^ We also calculated the dispersion energy contributions to lattice energies using the DFT-D3 program of Grimme, with Becke–Johnson damping.^38,39^ We considered the basis set superposition error (BSSE) by the counterpoise method.^40^ The energy convergence of the optimization and energy calculation was set to 10^−7^ hartree. The root-mean-square (RMS) values for calculation convergence were set to 0.0003 and 0.0012 au for energy gradient and atomic displacement, respectively. All calculations were conducted on a Linux cluster.

#### 2.7.3 Hirshfeld surface analysis

We performed Hirshfeld surface analyses^41,42^ with CrystalExplorer (Version 3.1)^43^ to account for the relative contributions to intermolecular interactions by various molecular contacts in the polymorphs.

## 3. Results and discussion

### 3.1. Crystal structures

**Fig. 2:**
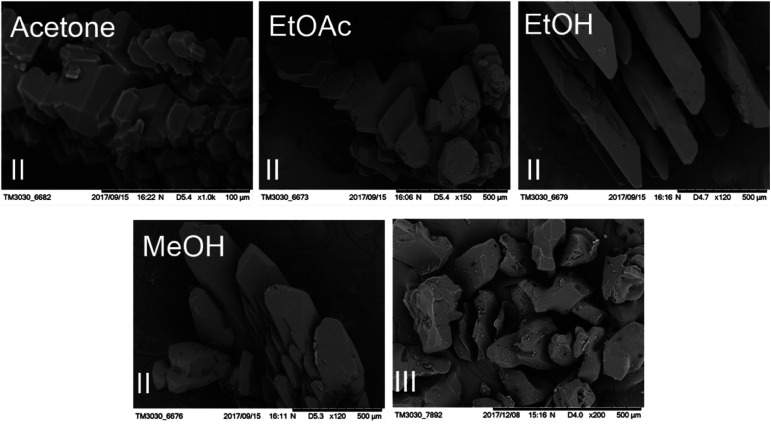
SEM micrographs of crystals of HDMPA form II from different solvents and form III.

**Fig. 3:**
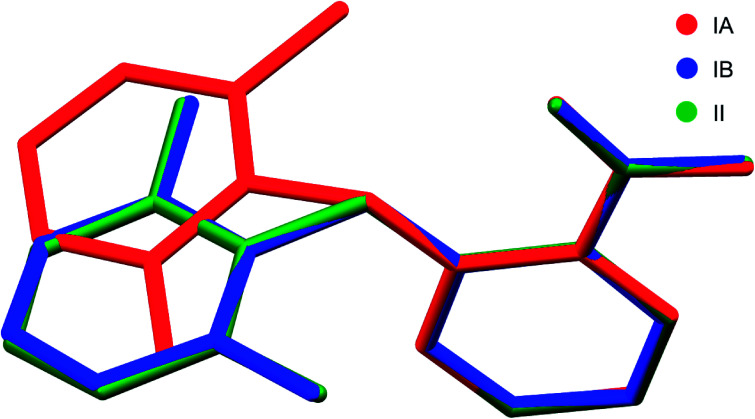
Superposition of all three molecular conformations in the asymmetric units of forms I and II of HDMPA.

Two new polymorphs (II and III) of HDMPA have been discovered in this study (Fig. 2). Form II crystals were grown as colorless blocks from (but not limited to) acetone, and form III was generated upon thermal treatment of form II samples. Form I crystals were obtained as colorless plates from a mixture of THF and acetone.^24^ Structure determination by single-crystal X-ray diffraction found form II to be triclinic, space group *P* (*Z* = 2), and form I was reported to be triclinic, space group *P* (*Z* = 4). No single crystal structure was determined for form III. Crystallographic data for form I and two structures of form II are given in Table 3; for complete CIF files, see the ESI.† The crystallographically independent conformations in each of the asymmetric units are slightly different, as suggested by the dihedral angle between the two aromatic rings (88.25(38)° for I-A and 86.69(34)° for I-B in form I, 88.91(4)° for II in form II). Conformational variability is also shown by a superposition of all three experimental conformations (Fig. 3).

**Table 3:** Crystallographic data of HDMPA polymorphs I and II (LT, low temperature, determined at 90 K; and RT, room temperature, determined at 296 K)

	I	II (LT)	II (RT)
Formula	C_15_H_15_NO_2_	C_15_H_15_NO_2_	C_15_H_15_NO_2_
Formula weight	241.28	241.28	241.28
Crystal size (mm)		0.50 × 0.50 × 0.15	0.30 × 0.20 × 0.20
Crystal system	Triclinic	Triclinic	Triclinic
Space group	*P*1̄	*P*1̄	*P*1̄
*a*/Å	15.8375(16)	7.4127(1)	7.524(2)
*b*/Å	7.5311(7)	8.0298(1)	8.103(2)
*c*/Å	11.1845(12)	10.9583(2)	11.191(3)
*α*/°	83.728(9)	73.3544(8)	72.582(4)
*β*/°	104.806(9)	83.3366(8)	83.740(4)
*γ*/°	79.038(8)	73.6078(8)	73.854(2)
*Z*, *Z*′	4, 2	2, 1	2, 1
*V*/Å^3^	1248.560	599.091(16)	625.1(3)
*D*_cal_/g cm^−3^	1.284	1.338	1.276
*T*/K	296	90(2)	296(2)
Abs. coeff. (mm^−1^)		0.089	0.085
*F*(000)		256	254
Range (deg)		1.94–27.49	1.91–25.00
Limiting indices		−9 ≤ *h* ≤ 9	−6 ≤ *h* ≤ 8
		−10 ≤ *k* ≤ 10	−9 ≤ *k* ≤ 9
		−14 ≤ *l* ≤ 14	−13 ≤ *l* ≤ 12
Completeness to 2*θ*		99.5%	98.5%
Unique reflections		2737	2170
*R*_1_[*I* > 2*σ*(*I*)]	0.0553	0.0434	0.0490
w*R*_2_ (all data)	0.0553	0.1190	0.1552

**Fig. 4:**
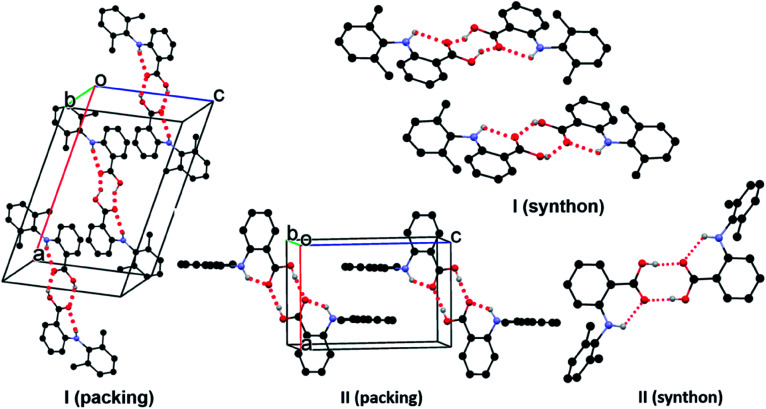
Crystal packing of I and II. For clarity, only hydrogens involved in hydrogen bonds (dotted line) are shown.

In form I, the asymmetric unit consists of two molecules (*Z*′ = 2), each having a twisted conformation. The two molecules form an acid–acid dimer hydrogen-bonding motif (R22(8)).^44–46^ The intermolecular hydrogen bond has a bond distance and bond angle of 1.888 Å and 138.28° for O16–H16⋯O15 for molecule A, and 1.830 Å and 140.31° for O16–H16⋯O15 for molecule B. In addition to the intermolecular hydrogen bonds, an intramolecular hydrogen bond exists between the carboxylic acid carbonyl O and the NH bridging the two aromatic rings, with a bond distance of 2.140 Å and bond angle 119.94° for molecule A and 2.071 Å and 120.79° for molecule B (Fig. 4).

Form II has one molecule in the asymmetric unit (*Z*′ = 1), and the molecule shows a twisted conformation, with the aromatic rings being perpendicular, as suggested by a dihedral angle of 88.91(4)°. The molecules form centrosymmetric acid–acid dimers. The intermolecular hydrogen bond has a bond distance and bond angle of 1.684 Å and 174.34° for O16–H16⋯O15. Both the bond distance and bond angle are significantly different from those in form I. The intramolecular hydrogen bond between the carbonyl O of the carboxylic acid and the anilino NH has a bond distance of 2.020 Å and bond angle of 135.16°. The intramolecular hydrogen bond parameters are also different from those in form I.

An exhaustive polymorph screening was performed to obtain the literature form I, yet only the new form II was generated. Questions were raised on the authenticity of the literature form. Simulated PXRD patterns are almost identical between forms I and II. But disappearing polymorphism could be the reason of the absence of form I.^47^

### 3.2. Thermal properties

**Fig. 5:**
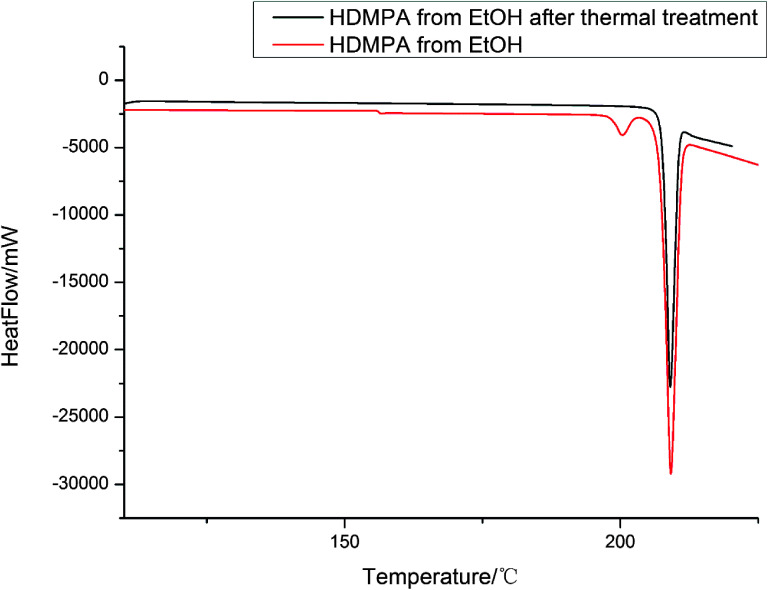
DSC thermograms of HDMPA form II before and after thermal treatment.

DSC was conducted to investigate the thermal properties of form II, shown in Fig. 5. Form II has two endothermic DSC peaks. The first, with an onset temperature of 199.2 °C, appears to be a phase transition to a new form that melts at approximately 201.6 °C. Based on the DSC thermograms, we can infer that form II converts into a third form when heated. As mentioned before, form I has *Z*′ = 2 and form II has *Z*′ = 1 and most of the time, the *Z*′ = 1 form is more stable than the high *Z*′ forms,^48,49^ so it is unlikely that form II transforms into form I, suggesting that the new form (labelled III above) could be a third form.

**Fig. 6:**
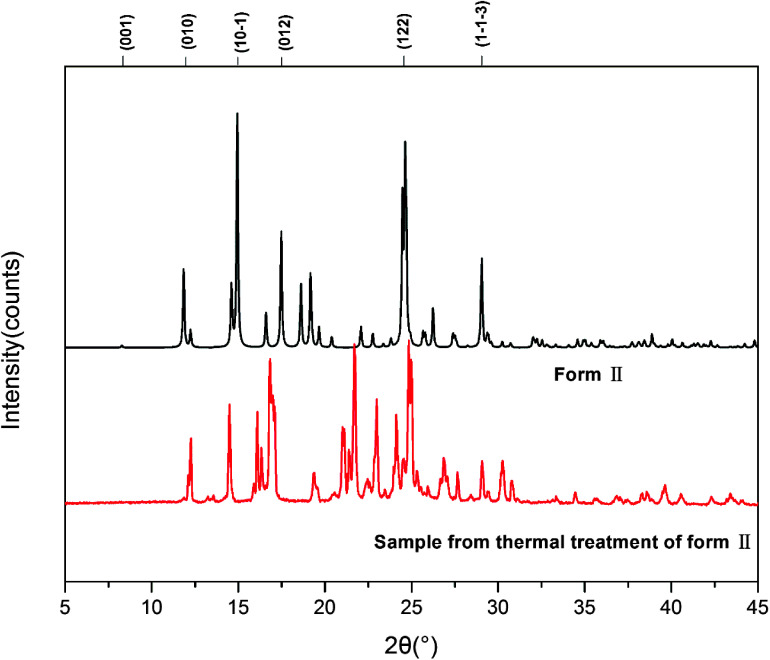
PXRD patterns of HDMPA form II before and after thermal treatment.

We also investigated the reversibility of the phase transition. When the sample was heated to a temperature just below the melting (200 °C) and then cooled down to room temperature, the DSC of the new sample showed only one peak, which corresponds to the melting of the sample. PXRD was measured for form III. The patterns of form III is different from that of form II as shown in Fig. 6.

**Fig. 7:**
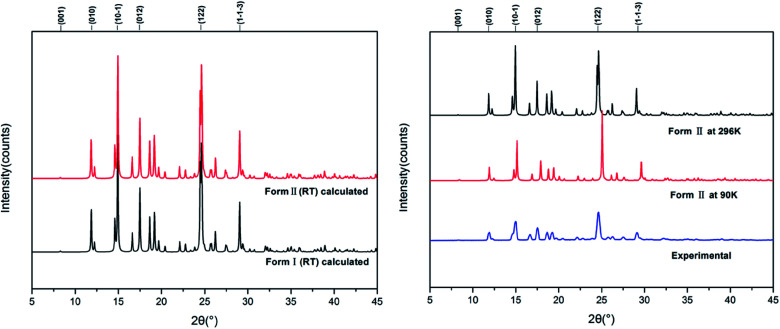
Experimental and calculated PXRD patterns of HDMPA polymorphs.

Fig. 7 shows the calculated powder X-ray diffraction patterns of forms I and II collected at room temperature (a), along with the matching of PXRD patterns calculated from the single-crystal structures determined at both 90 K and 296 K and the experimental PXRD patterns. The calculated PXRD patterns of the literature form and the new form are nearly identical, and the experimental and calculated PXRD patterns for form II match extremely well.

#### 3.2.1 Spectroscopic characteristics

**Fig. 8:**
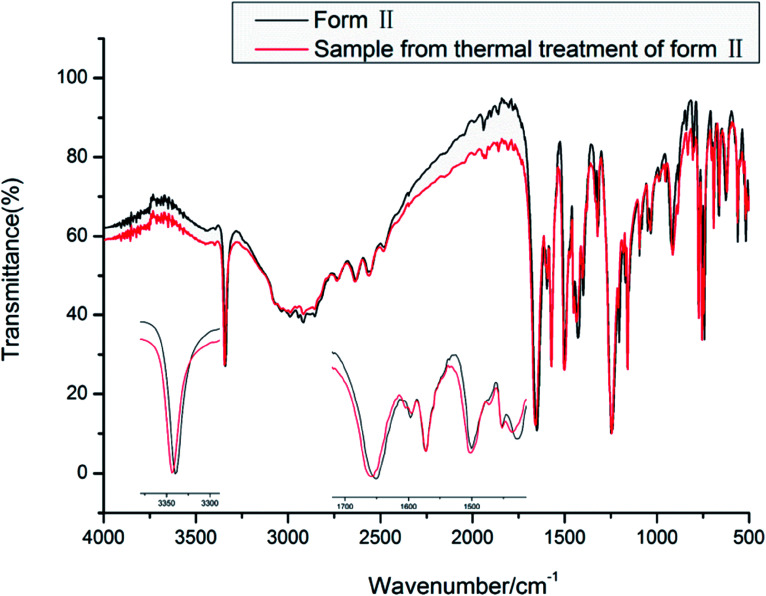
IR spectra of the new forms of HDMPA.

IR spectra of the two new forms of HDMPA are shown in Fig. 8. They show similar IR spectra, but subtle differences are observed as indicated in Table 4.

**Table 4:** Characteristic IR peaks of the new forms of HDMPA

	N–H stretch	Carboxyl O–H stretch and aromatic/aliphatic C–H stretch	Carboxyl C=O stretch	Aromatic C=C stretch
Form II	3339.5	3031.7–2565.3	1650.8	1596.6
				1572.9
				1500.1
Form III	3343.3	3077.6–2561.0	1658.4	1595.0
				1572.5
				1501.8

The vibrational spectra show the effect of the intramolecular hydrogen bond between NH and O=C. The NH stretch is at a lower frequency of around 3339.5 and 3343.3 cm^−1^ for forms II and III, respectively. The carboxyl C=O stretch is at 1650.8 and 1658.4 cm^−1^ for forms II and III. The carboxyl O–H and aromatic/aliphatic C–H as well as aromatic C=C stretches show subtle differences between forms II and III as indicated in Table 4.

**Fig. 9:**
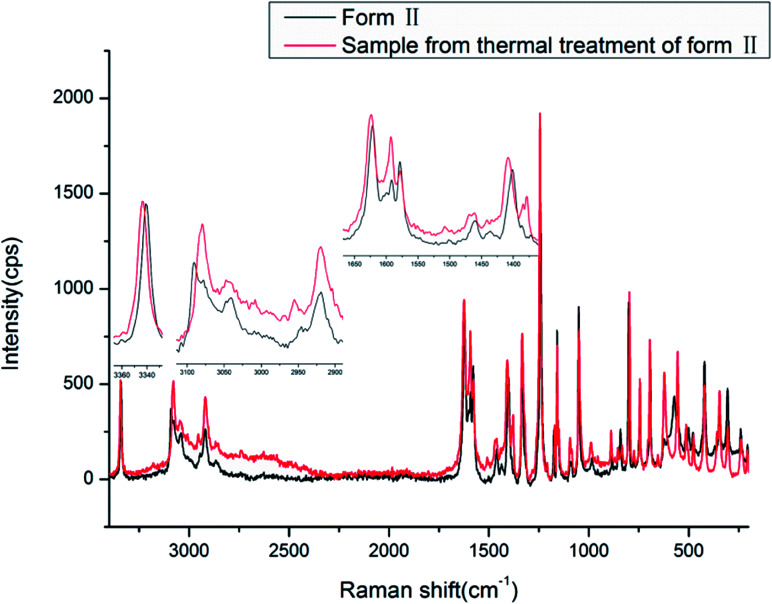
Raman spectra of HDMPA polymorphs.

The Raman spectra of the two new forms of HDMPA were measured for comparision (Fig. 9). Subtle yet distinguishable differences are observed (Table 5).

**Table 5:** Characteristic Raman peaks of the new forms of HDMPA

	N–H stretch	Carboxyl O–H stretch and aromatic/aliphatic C–H stretch	Carboxyl C=O stretch	Aromatic C=C stretch
Form II	3340.6	3090.5–2919.0	1621.5	1591.4
				1578.3
				1401.2
Form III	3343.4	3079.4–2919.4	1623.7	1592.5
				1578.0
				1408.2

The Raman spectra also show the effect of the intramolecular hydrogen bond between NH and O=C. The NH stretch is at a lower frequency of around 3340.6 and 3343.4 cm^−1^, and the carboxyl C=O stretch is at 1621.5 and 1623.7 cm^−1^ for forms II and III, respectively. The carboxyl O–H and aromatic/aliphatic C–H as well as aromatic C=C stretches also show subtle differences between forms II and III as indicated in Table 5.

### 3.3. Computational results

For forms I and II, calculated lattice energies based on the empirically augmented DFT method are −48.14 and −50.31 kcal mol^−1^. The energy difference between forms I and II is 9.07 kJ mol^−1^, slightly higher than 6 kJ mol^−1^, which is in agreement with the observation that conformational polymorphs have higher lattice energy differences.^50^ Two forms have similar calculated densities at 296 K (I: 1.284 g cm^−3^ and II: 1.276 g cm^−3^). While form I is slightly denser than form II, its lattice energy is higher than that of form II, which might be due to the hydrogen bond difference between the two forms, since form II has a shorter and more linear hydrogen bond than form I. Due to the uncertainty inherent to the computational methods employed (which may range from a few kJ mol^−1^ to a few kcal mol^−1^, or even higher), the small energy difference between forms I and II should not be regarded as evidence of which is more stable. Also it should be pointed out that discrepancy could arise since the computation implicitly assumed a temperature of 0 K, whereas the densities are based on the structures measured at 296 K, and the DSC experiments were performed at elevated temperatures.

**Fig. 10:**
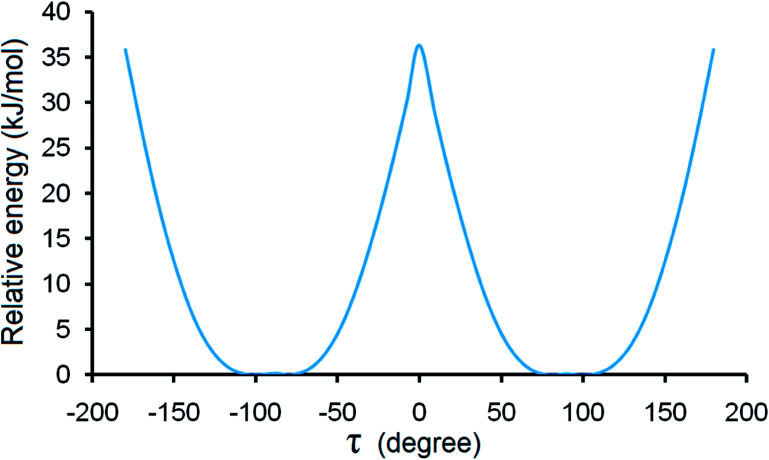
Conformation scan of single HDMPA molecule.

Although steric hindrance can limit the flexibility of HDMPA, polymorphism could arise from its intrinsic conformational flexibility. The conformational scan conducted over *τ* for a single HDMPA molecule is illustrated in Fig. 10. The global minima of *τ* are identified at ±78.5° and ±98.0° due to the symmetry of the benzene ring. The conformers of both forms are located near the minima, but in two different energy valleys. Thus it appears that the polymorphic system is one of conformational polymorphism, not merely due to conformational adjustment.^51^ The torsion angle of all three conformations is listed in Table 6.

**Table 6:** Torsion angle of the conformers

Conformer	Torsion angle (C6–N7–C8–C9/C13) (°)
I-A	−102.32/79.77
I-B	94.88/−79.51
II	79.63/−99.50

#### 3.3.1 Hirshfeld surface analysis

**Fig. 11:**
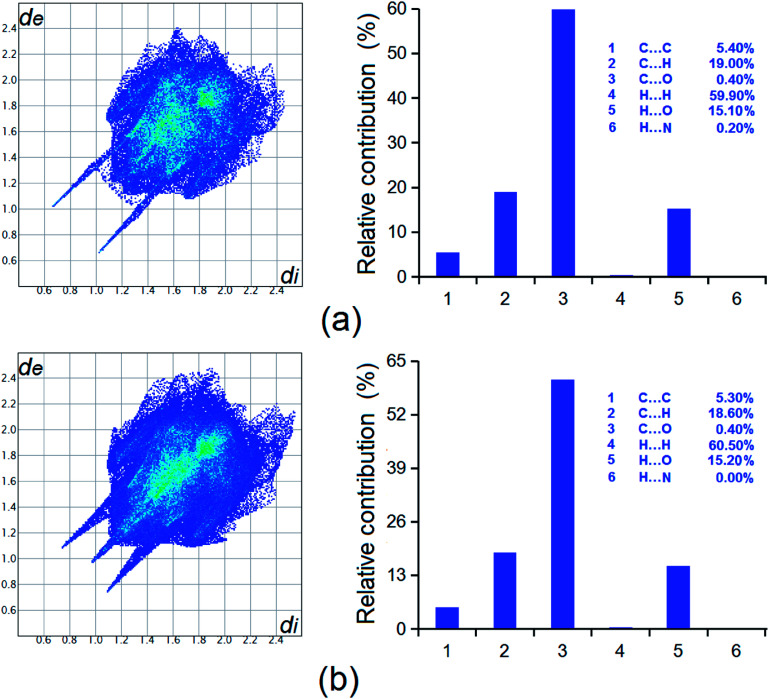
Hirshfeld surface analysis of the two forms of HDMPA, (a) form II; and (b) form I.

As shown in Fig. 11, the Hirshfeld analysis results indicate that in all six potential molecular contacts, hydrogen–hydrogen contacts predominate, contributing 60.5 and 59.9% of the overall intermolecular interactions in forms I and II, respectively. The second most significant intermolecular interaction is C⋯H, resulting in 18.6 and 19.0% of the sum of intermolecular interactions in the individual forms. In addition, H⋯O interactions also contribute significantly to the total intermolecular interactions, being 15.2 and 15.1% in forms I and II. Taken together, the crystal structures were stabilized by a variety of interactions.

## 4. Conclusions

Two new forms have been discovered for HDMPA, a fenamic acid with potential as an NSAID and in treatment of thyroid hormone disorders. Form II was characterized by structure determination by single-crystal X-ray diffraction, PXRD and spectroscopic methods such as FT-IR and Raman. The polymorphism appears to result from the conformational flexibility of the molecule, as suggested by the conformers in the two modifications and the conformational scan. The phase behaviors of the new forms were studied by DSC. The metastable form II appears to convert into form III on the application of thermal energy. Form III was characterized by PXRD, FT-IR and Raman spectroscopy. Lattice energies were calculated to be −48.14 and −50.31 kcal mol^−1^ for forms I and II suggesting the relative stabilities of the polymorphs, and Hirshfeld analysis indicated intermolecular interactions such as H⋯H, C⋯H, and H⋯O contribute significantly to the overall stability of the forms. This study clearly indicates that thermal treatment of crystalline samples could be an efficient approach for new polymorph generation. We are currently attempting to obtain single crystals for form III.

## Conflicts of interest

There are no conflicts to declare.

## Supplementary Material

RA-008-C7RA13353G-s001
