# Correction: Solution growth and thermal treatment of crystals lead to two new forms of 2-((2,6-dimethylphenyl)amino)benzoic acid

**DOI:** 10.1039/d0ra90012e

**Published:** 2020-01-27

**Authors:** Rong Hu, Yunping Zhoujin, Meng Liu, Mingtao Zhang, Sean Parkin, Panpan Zhou, Jianzhi Wang, Faquan Yu, Sihui Long

**Affiliations:** Key Laboratory for Green Chemical Process of Ministry of Education, School of Chemical Engineering and Pharmacy, Wuhan Institute of Technology 693 Xiongchu Road Wuhan Hubei 430073 China fyuwucn@gmail.com sihuilong@wit.edu.cn longsihui@yahoo.com +86 02787194980; Computational Center for Molecular Science, College of Chemistry, Nankai University Tianjin China; Department of Chemistry, Lanzhou University Lanzhou Gansu China; Department of Chemistry, University of Kentucky Lexington Kentucky USA

## Abstract

Correction for ‘Solution growth and thermal treatment of crystals lead to two new forms of 2-((2,6-dimethylphenyl)amino)benzoic acid’ by Rong Hu *et al.*, *RSC Adv.*, 2018, **8**, 15459–15470.

The new form, *i.e.* form II of 2-((2,6-dimethylphenyl)amino)benzoic acid identified in the original article is the same form as the one reported by V. Dokorou *et al.*^1^ The crystal structure reported by Dokorou is incorrect as they mistakenly doubled the unit cell, leading us to believe we were reporting a new form.

The recently published Comment article^2^ highlights our error in reporting form II as new.

The Royal Society of Chemistry apologises for these errors and any consequent inconvenience to authors and readers.

## Supplementary Material
